# Given the Cold Shoulder: A Review of the Scientific Literature for Evidence of Reptile Sentience

**DOI:** 10.3390/ani9100821

**Published:** 2019-10-17

**Authors:** Helen Lambert, Gemma Carder, Neil D’Cruze

**Affiliations:** 1 Animal Welfare Consultancy, 11 Orleigh Cross, Newton Abbot, Devon TQ12 2FX, UK; 2 Brooke, 2nd Floor, The Hallmark Building, 52-56 Leadenhall Street, London, EC3M 5JE, UK; gemmacarder@yahoo.co.uk; 3 World Animal Protection, 5th Floor, 222 Gray’s Inn Rd, London WC1X 8HB, UK; NeilDCruze@worldanimalprotection.org; 4 The Wildlife Conservation Research Unit, Department of Zoology, University of Oxford, The Recanati-Kaplan Centre, Tubney House, Abingdon Road, Tubney OX13 5QL, UK

**Keywords:** animal welfare, reptile, sentience, cognition, emotion, tortoise, turtle, lizard, snake, exotic pet trade

## Abstract

**Simple Summary:**

Reptiles are popular pets around the world, although their welfare requirements in captivity are not always met, due in part to an apparent lack of awareness of their needs. Herein, we searched a selection of the scientific literature for evidence of, and explorations into, reptile sentience. We used these findings to highlight: (1) how reptiles are recognised as being capable of a range of feelings; (2) what implications this has for the pet trade; and (3) what future research is needed to help maximise their captive welfare. We found 37 studies that assumed reptiles to be capable of the following emotions and states; anxiety, stress, distress, excitement, fear, frustration, pain, and suffering. We also found four articles that explored and found evidence for the capacity of reptiles to feel pleasure, emotion, and anxiety. These findings have direct implications for how reptiles are treated in captivity, as a better understanding of their sentience is critical in providing them with the best quality of life possible.

**Abstract:**

We searched a selection of the scientific literature to document evidence for, and explorations into reptile sentience. The intention of this review was to highlight; (1) to what extent reptile capability for emotions have been documented in the scientific literature; (2) to discuss the implications this evidence has for the trade in reptiles; and (3) to outline what future research is needed to maximise their captive welfare needs. We used 168 keywords associated with sentience, to search through four journal databases and one open-access journal. We recorded studies that explored sentience in reptiles and those that recognised reptile sentience in their experiments. We found that reptiles were assumed to be capable of the following emotions and states; anxiety, distress, excitement, fear, frustration, pain, stress, and suffering, in 37 articles. We also found four articles that explored and found evidence for the capacity of reptiles to feel pleasure, emotion, and anxiety. These findings show that reptiles are considered to be capable of experiencing a range of emotions and states. This has implications for how reptiles are treated in captivity, as a better understanding could help to inform a range of different operational initiatives aimed at reducing negative animal welfare impacts, including improved husbandry and consumer behaviour change programmes.

## 1. Introduction

Generally, it is accepted that all vertebrates are sentient beings [1,2], but the lack of consideration for reptiles in legislation and practice suggests that their capacity to feel may not be fully understood, and thus their sentience not widely accepted [3]. Attitudes towards different species, and belief in their capacity to suffer, is influenced by several factors [4]. For example, how people perceive different species’ capacity for sentience is thought to be directly related to how much they differ phylogenetically from humans [3,5]. This, along with unfamiliarity with the taxa [6], puts reptiles at a distinct disadvantage, compared with mammalian species such as dogs and cats [7]. Attitudes towards reptile sentience are important for how pet reptiles are treated. There are concerns that if an owner attributes little to no degree of sentience to their pet, they may be less likely to be concerned with their welfare, as they will not believe that they can feel fear, pain, or pleasure, or that their ability to do so matters [3]. As a result, this can affect an owner’s motivation to treat that animal well, to prevent negative states, or to promote positive ones. Consequently, many pet owners may be unaware that they are causing their pet to suffer unduly, and education regarding both their pets capacity to suffer, and how to meet their welfare needs is required [3]. Claims that reptiles do not need space, or require complex environments, or compared to birds and mammals, possess only basic ways of interacting with their environments, can be used as justifications for keeping reptiles in minimalist vivariums that are too small for their needs, and offer little positive stimulation that would occur in their natural environment, and has led to the criticism in the trade of reptiles [8].

Reptiles are far more complex than some people realise. For example, some species are highly social, although sociality, in general, is increasingly recognised in reptiles; popular claims of behavioural sedentarism are exaggerated, and many species manifest extensive natural home ranges, thus minimum spatial provisions are implicitly problematic, and in general, reptiles may be more aware of their environments and the limitations of those conditions than many observers believe [7,8,9,10,11,12,13]. Therefore, the perceptions’ of reptiles that underestimates them as being unintelligent and basic in their animal welfare needs, can mean that they suffer considerably in captivity.

Reptiles are a popular pet around the world, with ownership likely to consist of tens of millions of animals, if not more [7]. Accurate numbers of the trade in exotic pets are unavailable due in part to much of it involving illegally wild-caught animals [8]. In the UK, however, between 2018–2019, there were thought to be around 1.7 million reptiles kept as pets in homes [14]. Whereas, in 2017–2018, the USA was thought to have 9.4 million reptiles as pets [15]. The growing demand for reptiles has led to an increase in their removal from the wild, and an increase in captive-bred operations, both of which have considerable welfare implications for the animals involved [16,17,18].

To help maximise reptile welfare in captivity, an understanding of the landscape of reptile sentience research is required, as is the need to identify areas of strength and clarity, as well as areas where more research is required. In this review, we have sought to explore the scientific literature regarding reptile sentience within the last 20 years (1999–2018). Specifically, we have searched through four journal databases and one open-access journal to find research articles that are exploring or assuming the capacity for sentience in reptiles. To do this, we have used 168 keywords, which describe various aspects of sentience. For this review, we have defined sentience as the capacity of an animal to feel and experience both positive and negative emotions and states [1]. These feelings may range from basic, but important states, such as pain and fear, to more complex emotions, such as grief and empathy [19]. Emotions are a core component of sentience, and they form the majority of the sentience keywords we have used (see Table A1). Emotions can be defined as short-lasting states that vary in valence from positive to negative, and in the degree of associated arousal (high to low) [20]. Personality was not included within this definition of sentience, as although an individual’s personality can affect how they cope with their environment, the possession of personality traits does not have any bearing on whether they can consciously experience emotions [21,22].

The aims of this review were to (1) assess the extent to which reptile sentience features in a selection of the scientific literature, (2) to assess which aspects of sentience have been studied and in which reptilian taxa, and (3) to suggest recommendations for future research in this regard.

## 2. Methods

The literature review was carried out in two stages. First, we searched the literature for evidence of sentience. Second, we searched the journals, identified in phase one, for generic reptile studies as a source of comparison.

### 2.1. Phase One

#### 2.1.1. Keywords

To search for evidence of sentience in the scientific literature, we used a list of 168 of the keywords which, referred to traits and aspects of animal sentience (Table A1). The keywords had previously been used for review of sentience research in Proctor et al. (2013) [23]. Nine of the keywords used in the 2013 review were deemed inappropriate for this study, as they were focused on aspects of personality and cognition, and not sentience, and so were not used.

#### 2.1.2. Literature Search

We used the keywords to search through four journal databases (ScienceDirect, BioOne, Ingenta Connect, and MDPI) and one open-access journal (PlosOne) for the occurrence of each of the 168 keywords in association with the word ‘reptile’ in the abstract, title or keywords, using the Boolean operator AND. Within those databases, we searched for all research articles published between 1999 and 2018. We chose this period because it allowed for a large and recent study period, yet it was still feasible given our time restraints. Each returned article’s entire text was searched manually. Each returned article was then reviewed individually to ensure that the keyword was used within the correct context. The keyword had to refer to the animal’s subjective emotional state. For example, ‘distress’ had to refer to emotional distress, and not to physiological distress. For instance, a reference to respiratory distress, without mentioning any emotional component or suffering, would not have been included. Furthermore, the keyword had to be used in reference to the reptile species studied in the article. This meant that the returned articles were experimental studies, which either explored that species’ capacity for the keyword, or were assuming their capacity for it in their experiment. For example, studies exploring the species capacity for the keyword ‘pain’, may include an exploration into whether the species could feel pain. Whereas a study assuming the capacity for pain in the reptile species studied may be looking for signs of pain when testing the effectiveness of an analgesic. If the article just referred to the keyword in reference to another study’s findings, and not in relation to the species they were testing, it was not included as a result. For each returned article, we recorded the following data; journal, publication year, species studied, and whether the keyword was explored or assumed.

### 2.2. Phase Two

To determine what proportion of the reptile literature the sentience articles represented, we further explored the 17 journals that had returned results. We searched each of the journals for the word ‘reptile’, to determine how many general reptile research articles they had published in total during the 1999–2018 study period. These searches were performed on the article’s title, abstract and keywords, and these were checked to verify that the article was utilising a reptile species in their study by reviewing their title, or abstract.

#### 2.2.1. Inter-Rater Reliability Tests

Two of the authors collected the data, and both had previously performed a similar systematic review using many of the same keywords [23]. To ensure consistency, the definitions and working examples were used from the previous review [23]. In addition, both researchers conducted three inter-rater reliability tests before, mid-way, and after the data collection period. For each of these tests, both researchers reviewed the same six articles and recorded whether or not the keyword was used correctly for each, and whether it was explored or assumed. Each test used three randomly selected keywords, and a different selection of six articles were used for each test. The lead researcher’s analyses served as the silver standard throughout training and for all comparisons. The researchers’ responses were then compared to one another, and a percent agreement was calculated by dividing the number of agreement scores by the total number of scores. Test one, two, and three, all returned a 100% agreement score.

#### 2.2.2. Comparison with Data on Mammals

To provide context for the results, and to position knowledge of reptile sentience in relation to a well-studied taxon, we compared the results with those from a review that explored mammal sentience [23]. The 2013 review used 169 of the same keywords as the current review, but was performed on a different time-frame (1990–2011), and only on two journal databases (Science Direct and Ingenta Connect).

### 2.3. Data Analysis

Descriptive analyses were performed on the returned articles.

## 3. Results

Of the 168 keywords searched for, only 10 returned results (see Table 1 and Table A2). These keywords were found in 41 articles, three of which featured more than one keyword, leaving 38 individual papers.

### 3.1. Support for Reptile Sentience

#### 3.1.1. Articles Assuming Reptile Sentience

Eight different sentience traits/aspects were assumed to exist in reptiles in the literature reviewed (according to the eight key words returning results) (see Table A2). These were; anxiety (three articles), distress (two articles), excitement (one article), fear (three articles), frustration (one article), pain (22 articles), stress (four articles), and suffering (one article). These keywords were found in a total of 37 articles.

#### 3.1.2. Articles Exploring Reptile Sentience

The following three sentience keywords were explored by researchers in the literature that we reviewed; anxiety, emotion, and pleasure. The keyword emotion was explored in two different articles, and the keywords; anxiety and pleasure, were explored in one article each. The keywords pleasure and emotion were only explored in reptiles and were not assumed to exist already. All four articles successfully found evidence for the capacity of anxiety, emotion, and pleasure in the reptile species they studied, apart from one study which found evidence for anxiety in red-footed tortoises, but only tentative findings for bearded dragons (see Table 1). Table 1 provides a summary of how each of the returned articles used the keyword.

### 3.2. Comparison with Mammals

We compared the number of keywords with returned results with those from a similar review performed on mammals (Table 2) [23]. Of the 168 keywords used in both studies, the 2013 review had 35 keywords return results, whereas the current reptile review returned 10. All of the keywords that returned results for reptiles also returned results for mammals.

The 2013 review found that 74% of the mammal articles arose from just five top keywords; fear, stress, pain, anxiety, and depression. Four out of these five words were also in the top five keywords for the current reptile review, although in a slightly different order; pain, stress and anxiety (joint second), and fear (Table 2).

### 3.3. Reptile Species Studied

A total of 50 reptile species were studied in the returned articles, representing 0.46% of the known 10,793 reptile species currently identified [28]. Overall, the most common order of reptiles studied was the Squamata order (80%), followed by Testudines (14%), and then Crocodilia (6%). The fourth reptile order, Sphenodontia, was not represented, but as this order is only comprised of two species, this was expected. Twenty-two reptile families were included in the study sample, and the top five were; Scincidae (eight species), Gekkonidae (seven species), Lacertidae (five species), Colubridae (four species) and Emydidae (three species). The sentience keywords were assumed in 46 different species and were explored in eight different species (see Table 3).

Of the 50 species covered in the reviewed literature, 64 of them were featured once. The species that were studied more than once in different articles are shown in Table 4.

### 3.4. Publication Years

**Figure 1:**
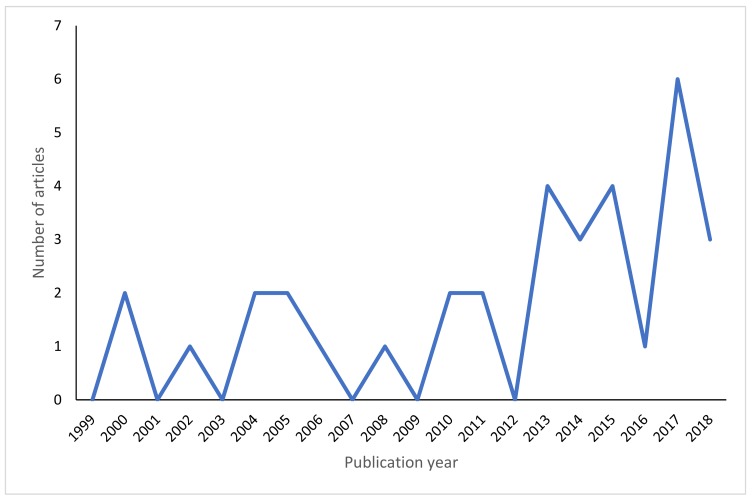
Number of sentience papers published between 1999–2018.

We searched for articles published between 1999 and 2018, and the number of articles published each year can be seen in Figure 1. The number of articles returned for the keywords shows a slight increase in recent years, although Figure 1 shows that this is not a steady increase.

### 3.5. Scientific Sources

**Figure 2:**
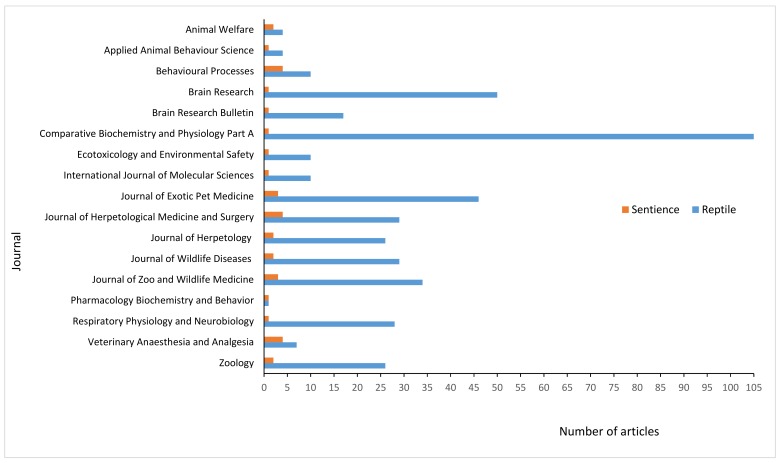
The total number of returned articles exploring and assuming sentience in reptiles between 1999 and 2018 from five sources (Science Direct, Ingenta Connect, PLOS ONE, BioOne, and MDPI), and the total number of articles featuring the word ‘reptiles ‘in the abstract, title, or keywords for each journal.

All of the returned articles came from 17 individual journals, from five different sources (ScienceDirect, BioOne, Ingenta Connect, MDPI, and PlosOne). We also calculated how many research articles each of these journals published on reptiles in general, between 1999 and 2018. The comparison between these findings can be seen in Figure 2.

## 4. Discussion

The science of animal sentience is still relatively new, and attention in the published literature in this area is steadily increasing [23]. Nevertheless, this review has shown that acceptance for several aspects of reptile sentience is present in the recent published scientific literature, and furthermore, it has featured on numerous occasions as part of their experimental use. In this review, we found that reptiles were assumed to be capable of at least eight different aspects of sentience in the scientific literature; anxiety, distress, excitement, fear, frustration, pain, stress, and suffering. Furthermore, four studies also explored and found evidence of anxiety, emotion and pleasure in reptiles. Sentience is not, however, a major area of focus in the published reptile-oriented scientific literature. In the sources reviewed, we found that 17 journals published articles that assumed or explored reptile sentience in the 20-year study period. The sentience articles only represented a small proportion of the research articles that these journals published on reptiles in general. This means that the majority of studies using reptiles do not refer to their capacity for sentience. Of course, for many articles, sentience would not be relevant, and it was not in the scope of this review to explore what each of these studies was focused on. The fact, however, that the vast majority of studies on reptiles do not mention any of the 168 keywords related to sentience, does possibly highlight a lack of concern for the mental wellbeing of reptiles. Particularly, if we consider the relevance of certain keywords (e.g., pain and stress) to many experimental uses of animals.

There is already a recognised bias towards mammalian species that has shown persistence over the last 30 years or longer [23]. This has meant that taxa, such as reptiles, which do not feature as greatly in laboratories or on farms, or in the publics’ sphere of concern, have not received the scientific attention that they deserve. By nature, we humans are drawn towards other mammals, and are better able to empathise with, and accept sentience in mammalian species, than we are other taxa, primarily due to our familiarity with them and the similarities in behaviour and physiology [4,6,23]. These anthropocentric tendencies apply across all non-mammalian taxa. ‘Cold-bloodedness’ in fish has, for example, been commonly used as a reason for doubting their capacity for pain and other subjective experiences [29]. Such physiological or metabolic distinctions, however, are unrelated to an animal’s capacity for subjective states and have no place when it comes to assigning consideration for welfare and accepting sentience. Instead, we should be applying critical anthropomorphism when it comes to animal research. Uncritical anthropomorphism is unhelpful and can be damaging, as animals’ behaviour and needs can be misunderstood, and it risks weakening the scientific field of sentience [1,30]. Critical anthropomorphism, however, effectively uses our innate intuitions and empathy, along with objective evidence and an understanding of an individual animal and their species, to draw conclusions regarding sentience, and to steer research initiatives [30,31,32].

A total of 50 species of reptiles were featured in the returned articles, representing >1% of the 10,793 known reptile species, and a fraction of the 550 or more reptile species thought to be traded internationally [8,28]. Although this suggests that the literature on sentience does not represent reptiles fairly, this is not unique to this taxonomic group, as birds, invertebrates and fish are also under-represented in this regard [23]. For example, Proctor et al. found that species from the Mammalia class (mammals) were studied in 91.89% of the 2562 articles they reviewed, compared with 4.54% for Aves (birds), 3.66% for invertebrates, and 1.76% for Actinopterygii (bony fish). To provide context, the Mammalia class is comprised of 6495 species [33], Aves; around 10,000 species, invertebrates; approximately 1.3 million species [34], and Actinopterygii; around 27,000 species [35]. In the 2013 review, only 79 of the 6495 possible mammalian species were featured, representing 1.21% of the entire taxa, and so despite there being a clear bias towards mammals, mammals are also still understudied in terms of sentience [23,33].

### 4.1. Implications for the Reptile Pet-Trade

What are the implications of this review and the issues of reptile sentience in terms of the current treatment of these animals in the commercial trade and associated ownership of exotic pets? The commercial trade in reptiles is already known to present several challenging situations that can negatively impact on the welfare of the animals involved, irrespective of whether they are wild-caught or captive-bred. When wild-caught, individuals may be exposed to stressful physical handling and injuries during capture, and then potential further stress and considerable mortality rates from the subsequent transportation, storage and processing [7]. Estimated mortality rates for reptiles during capture from the wild range from 5% to 100%, and between 5% and 25% during captive breeding for captive-bred species [7,36]. According to Warwick (2014) [7], however, it is important to consider that even a 1% transport mortality rate is likely to refer to millions of animals, given the scale of the industry.

Captive breeding may not all involve the same issues associated with wild capture, but those involved are still subjected to unnatural conditions associated with intensive rearing, packaging, and transportation [7], that can impact negatively on their physical and mental well-being. For example, in one major exotic animal wholesaler, researchers found 80% of the invertebrates, amphibians, reptiles, and mammals to be sick, injured, or deceased [36]. In addition, nearly 3500 deceased or moribund animals, most of whom were reptiles, were discarded weekly, equating to a 72% mortality rate during an average 6-week stock turnover [36]. This high mortality rate may not be unique to this wholesaler and is considered by some industry representatives, to be an industry standard [36].

Once they arrive in pet shops, or homes as pets, reptiles are then provided with environments very different from those that they would experience in the wild, and potentially with misguided husbandry that fails to meet their basic welfare needs [3]. For example, the finding that green iguanas and turtles show an increase in heart rate when gently handled, and that this is an indication of emotional stress (see Table 1) [24,25], has implications for the care of these animals in captivity. Such stress may not be exhibited behaviourally, and so the average person handling a lizard or a turtle may be unaware of the emotional stress they are causing them. Captive-bred individuals should not be considered to be adapted to captivity, and commonly manifest behavioural and physical signs of captivity-stress [37].

There has also been little research into the effects of selective breeding on reptiles, and there can be considerable welfare implications of selecting for certain traits, such as colours and patterns. In the ball python (*Python regius*), for example, selecting for the “spider morph” phenotype has led to an increase in a heritable neurological disorder referred to as the “wobble syndrome” [38]. This causes the snake’s head to wobble from side to side, and to occasionally flip backwards and upside down. In an assessment of the animal welfare impacts of this condition, one of the articles we reviewed discussed how the condition caused considerable frustration in the snakes during feeding [38]. Not only is this an acknowledgement of the capacity for snakes to suffer mentally from emotional states such as frustration, but it also highlights an increasing welfare issue for ball pythons, which is commonly viewed as an acceptable side-effect by pet owners and breeders.

Unlike many invertebrates, reptiles are protected under welfare legislation around the world, and so the acknowledgement that reptiles can suffer is recognised at a formal level [1]. Understanding what reptiles are capable of in terms of emotional states, however, still remains a useful basis for making decisions regarding their welfare [23]. For example, reptiles will often appear to be “thriving” physically in poor conditions, when they are actually suffering considerably [7]. The slow metabolism of reptiles means that they can tolerate poor welfare for longer than a mammal could, but this means that they suffer for longer [37]. Given the potential for poor welfare that reptiles can experience as a result of their involvement in commercial trade, recognising their capacity to suffer, feel pain, stress, fear, and other important sentience traits, is crucial in both changing perspectives towards their needs, and in highlighting any inadequacies of the legislation and associated exotic pet industry.

### 4.2. How Can This Evidence Be Used to Improve Reptile Welfare?

There is already considerable evidence of negative animal welfare impacts on reptiles that can be used to assess their welfare, and to make practical changes and inform ethical choices regarding their suitability to captivity [7]. Reptile behaviour can be used to identify disease, injuries and stress in the same way that mammal behaviour can [7]. For example, one of the studies reviewed showed that changes in feeding behaviour were a reliable indicator of pain in the ball python (*Python regius*), as those in pain showed delayed feeding [39].

Unfortunately, indicative behaviours can be ignored in reptiles, or are not seen as signs of suffering [3]. This oversight is likely to be due to the observer’s perception of the capacity for reptiles to suffer. If observers were to see similar behaviours in mammals, it is assumed a proportion of observers would likely deduce suffering and make practical changes to improve the animal’s wellbeing. The behaviour of reptiles, fishes and invertebrates, however, are often judged differently to that of mammals, due to pre-existing perceptions and biases [29,40,41]. Generally, reptiles are also not liked as much as mammals, and this can influence how they are perceived [42]. For example, when asked to rate on a 10-point scale how much they liked 40 different species, participants rated lizards as 5.0, and pythons as 3.2 [42]. In comparison, chimpanzees were rated as 8.2, and elephants as 7.8. Even between reptile owners and reptiles, the bond with reptiles is much weaker than with other animal types [7]. It could be argued that such findings may encourage the notion that reptiles are inferior to mammals. It would be interesting to explore which factors positively influence people’s opinions of reptiles. In the meantime, objective reviews are critical in highlighting what is known about the sentience of reptiles, and to demonstrate that these animals are capable of experiencing emotions. Research that highlights the complex needs of reptiles can also be used by advocates working to improve their welfare in captivity. For example, Pasmans et al. [43] recommend that the development and maintenance of reptilian species-specific husbandry requirements are needed to ensure optimal welfare for captive reptiles.

### 4.3. Limitations

It is the nature of reviews such as this one, that some articles may be missed. We selected five different sources intended to cover a breadth of articles and to try to capture the sources where papers on sentience were likely to be published. Nevertheless, despite our best attempts, there will still be articles that are not featured in this review. This review still provides a useful representation of the current trends in a considerable proportion of the scientific literature and highlights what is known about reptile sentience, as well as the areas that need further attention.

#### Areas for Future Research

Non-human animal sentience is a relatively emergent field and generally understudied [23,44]. Much of the research to date has been performed on laboratory and farm animals, and certain taxa, such as reptiles, have not received much attention in comparison [45]. The science of sentience is growing, however [23], and future research can address some of the gaps in our knowledge. We found a slight increase in the number of articles discussing reptile sentience over recent years, although the numbers are too small to show a definitive trend. The numbers of reptiles in the international pet trade are also increasing annually [14,17,46], and so more research is required to keep up with this trend and to address the growing welfare implications of trading and keeping reptiles as pets.

This review found that the majority of studies were focused on the Squamata order (snakes and lizards). This may be due to the convenience of their size, in terms of their suitability as a research species, as it also represents considerably more species than the other orders [28], or because they are thought to be more popular as pets [14]. Further research should continue to explore the sentience and cognitive abilities of Squamata, as relatively little is still known about their emotional lives. Other reptile taxonomic groups are also in need of increased research attention. For example, Table 4 shows that nine of the 50 species studied were studied more than once. Furthermore, Testudines only represented a fifth of the species studied and given that they are also commonly traded and kept as pets [14,47], further research into their sentience is clearly needed.

Sentience and animal welfare science tend to focus on the experience of negative states, such as pain and fear, as these are more urgent in regards to providing adequate welfare, and for ensuring that animals are not unduly suffering [48,49] There is still much more to know about the capacity and relevance of negative states in reptiles, and so this should continue to be explored. Future research should, however, attempt to address the lack of understanding regarding reptile species’ capacity for positive emotions, and the importance of these to their welfare. An animal can only have good, or even adequate welfare, if negative emotions and experiences are minimised, and positive states are promoted [50]. Therefore, understanding which positive states are most relevant to reptiles, and how they can be promoted, should be a priority for future research. This review uncovered a considerable lack of knowledge regarding positive states in reptiles. This was expected to some extent, due to there being so few articles considering sentience in reptiles in the first place. Nevertheless, the imbalance should be a consideration for future research studies.

The keyword ‘play’ was one of the positive keywords we searched for that failed to return any results in this review. There is, however, evidence of play in reptiles, and so the lack of returned articles may be due to the sources searched, or the use of the search term ‘reptiles’ rather than a lack of existing literature. Reptiles are known to perform various types of play behaviour, from interacting with objects, playing tug of war, and various forms of water play in aquatic species [51,52]. For example, Dinets observed various crocodile species playing with pink flowers in the water noted that small, pink objects were particularly favoured by the crocodiles, and other available objects were ignored [53]. Others have observed captive crocodiles playing with cinder blocks provided in a captive setting [54]. Yet, often such play behaviour in reptiles can be missed, due to their movements and behaviours being much slower than what we are used to, and them spending long periods inactive [53,55]. For example, in his review of play behaviour in fish, frogs, and reptiles, Burghardt refers to how the play behaviour of Komodo dragons resembles play in dogs when it is filmed and sped up [51]. Consequently, we suggest that future reviews should use a wider range of play-associated language and other taxonomic terms relating to reptiles (e.g., crocodilians) to explore this area further.

In this review, we decided to focus our efforts on the field of sentience. The cognitive abilities of reptiles are, however, also grossly under-represented in the scientific literature [52]. Cognition can be defined as how animals perceive, process and retain information, including how they respond to such information [56]. Under this definition, cognitive processes including learning, perception, memory, and decision-making are included. We regard cognition as a separate entity from sentience, as how intelligent an animal is, ultimately, has no bearing on whether or not they can suffer [57]. We recognise that an animal’s cognitive ability can influence how well or poorly they cope with their environment, but the degree of cognitive ability is irrelevant to whether or not an animal can suffer [1]. Future reviews should consider exploring the evidence for cognitive abilities in reptiles, as highlighting both the emotional and cognitive capabilities of animals can be integral to their experience of captivity. This is particularly important for reptiles, as the general public generally perceive this taxonomic group as unthinking and unfeeling beings [3].

Further research should continue to explore what reptiles are capable of, both emotionally and cognitively. Future studies should also be ecologically relevant, and represent the natural lifestyles experienced by the species in question. Such information could be highly valuable in providing for their welfare. For instance, in existing captive conditions, reptiles may benefit from being cognitively stimulated [52]. An understanding of intelligence is also useful in showcasing these animals as thinking, feeling beings who matter, as opposed to automatic beings who are unaffected by poor treatment. For example, one study found that a turtle cognition research demonstration enhanced visitor engagement at a zoo, and improved keeper–animal relationships [58]. Alba et al. found that the demonstration increased the amount of time zoo visitors spent at the eastern box turtle (*Terrapene carolina carolina*) exhibit, which enhanced their chance to learn about the animals, and to understand how complex the species is. Furthermore, the keepers reported stronger bonds with the turtles, as a result of participating in the cognitive research sessions. These findings are important, as an improved bond with an animal, and a greater understanding of their mental state, can lead to improved treatment and welfare [3]. Furthermore, the demonstration appeared to be an indirect observation of the turtles, and so the turtles were not subjected to unnecessary and stressful handling, as seen in many animal–visitor interactions at zoos [59].

We chose not to include motivation in this review, although motivation and emotion are related, we do believe that they are distinguishable. We do recognise, however, that changes in emotional states can result in a change in motivation, likewise, changes in motivation can result in a change in emotional state. It could be beneficial, therefore, for future research to explore the extent to which reptilian motivation has been studied.

## 5. Conclusions

The scientific literature shows us that the capacity for reptiles to feel pain, stress, fear, and anxiety is accepted and utilised in scientific studies. Given how reptiles are sometimes poorly treated, however [8], and the general acceptance for potential suffering and high mortality rates in the pet trade [36], it is likely that this evidence is not always reaching those who care for captive reptiles [3], or that their long-held perceptions of reptiles clouds their judgment [3,7,60]. Furthermore, given the variation in physiological and behavioural adaptations to pain and suffering seen in reptiles, recognising changes in normal behaviour can be a challenge [61]. Research into the sentience of reptiles needs to continue to grow, and more importantly, the findings need to be communicated beyond the scientific community to the general public. The science of sentience can be used to engage the public with species and the welfare issues they face [1,62]. By showcasing the complex capacity for sentience that reptiles have, science can perhaps help position reptiles alongside the more popular mammalian species, and demonstrate that they can not only suffer, but that they are capable of other complex experiences and states [23,52,58]. If research can prove to the public that these sensitive, thinking, and feeling beings have a greater potential to suffer in poor captive conditions, then it could help inform a range of different operational initiatives aimed at reducing negative animal welfare impacts, including improved husbandry [3]. Such information can aid consumer behaviour change programmes, which aim to reduce the demand for exotic pets [63].

## Figures and Tables

**Table 1:** The articles found to explore sentience in reptiles, and a summary of the related findings.

Article	Keyword	Summary of Keyword’s Use
Cabanac, A., and Cabanac, M. (2000). Heart rate response to gentle handling of frog and lizard. *Behavioural Processes*, *52*(2–3), 89–95. [24]	Emotion	Green iguana’s (*Iguana iguana*) were handled to see whether they showed an increase in heart rate, indicative of emotional fever and the presence of emotion. They found that green iguanas have an emotional response to the stressful experience of handling.
Cabanac, M., and Bernieri, C. (2000). Behavioral rise in body temperature and tachycardia by handling of a turtle (*Clemmys insculpta*). *Behavioural Processes*, *49*(2), 61–68. [25]	Emotion	Turtles (*Clemmys insculpta*) were handled to see whether they showed an increase in heart rate, indicative of emotional fever and the presence of emotion. The resulting stress fever and tachycardia in the turtles were taken as signs of emotion.
Paradis, S., and Cabanac, M. (2004). Flavor aversion learning induced by lithium chloride in reptiles but not in amphibians. *Behavioural Processes*, *67*(1), 11–18. [26]	Pleasure	This article looked for flavour aversion learning in several reptile species (*Basiliscus vitattus*, *B. basiliscus*, *Eumeces schneideri*, *Mabuya multifasciata*). They found that the reptiles all showed flavour aversion learning, and they concluded that this may indicate that reptiles can experience sensory pleasure.
Moszuti, S.A., Wilkinson, A., and Burman, O.H.P. (2017). Response to novelty as an indicator of reptile welfare. *Applied Animal Behaviour Science*, *193*, 98–103. [27]	Anxiety	This article investigated the responses of red-footed tortoises (*Chelonoidis carbonaria*) and bearded dragons (*Pogona vitticeps*) to novelty to assess anxiety-like behaviour. They found different responses in the two species to novelty, and the authors concluded that the red-footed tortoises showed signs of anxiety in response to a novel environment, whereas the bearded dragon’s responses required further investigation.

**Table 2:** The number of returned results for the successful keywords searched for reptiles (current study), and for mammals from a previous review performed on the years 1990–2011 [23]. The entries highlighted in grey were the keywords which returned results for both mammals and reptiles.

Keyword	Number of Returns: Reptiles		Number of Returns: Mammals	
	Explored	Assumed	Explored	Assumed
Affective state	0	0	0	51
Agitation	0	0	0	13
Altruism	0	0	3	4
Anger	0	0	0	2
Annoyance	0	0	0	1
Anticipation	0	0	0	38
Anxiety	1	3	0	267
Apprehension	0	0	0	2
Arousal (emotional)	0	0	0	5
Boredom	0	0	0	4
Contentment	0	0	0	1
Depression	0	0	0	222
Despair	0	0	0	73
Disgust	0	0	0	2
Dislike	0	0	0	1
Distress	0	2	3	53
Eagerness	0	0	0	1
Emotion	2	0	0	16
Excitement	0	1	0	5
Fear	0	3	0	635
Frustration	0	1	0	24
Helplessness	0	0	0	98
Hostility	0	0	0	1
Joy	0	0	0	1
Nervousness	0	0	0	5
Optimism	0	0	1	0
Pain	0	22	2	303
Panic	0	0	0	43
Play	0	0	0	60
Pleasure	1	0	0	1
Stress	0	4	0	607
Suffering	0	1	0	15
Tenseness	0	0	0	3
TOTAL	4	37	15	2559

**Table 3:** The species studied in each of the returned articles. Species featured in more than one article for the keyword are marked.

Keyword	Assumed or Explored?	Species Studied
Anxiety	Assumed	Chinese lizard (*Eremias argus*); Red-eared slider (*Trachemys scripta elegans*); Wall lizard (*Podarcis muralis*)
Anxiety	Explored	Bearded dragon (*Pogona vitticeps*); Red-footed tortoise (*Chelonoidis carbonarius*)
Distress	Assumed	Baird’s ratsnake (*Pantherophis bairdi)*; Black ratsnake (*Pantherophis obsoletus*); Dune gecko (*Stenodactylus petrii*); Eastern glass lizard (*Ophisaurus ventralis*); Eastern hognose snake (*Heterodon platirhinos*); Eastern ribbonsnake (*Thamnophis sauritus*); Guinea legless lizard (*Lialis burtonis*); Green anole (*Anolis carolinensis)*; Gyldenstolpe’s worm skink (*Isopachys gyldenstolpei*); Gunther’s leaftail gecko (*Uroplatus guentheri);* Helmeted gecko (*Tarentola chazaliae*); Henkel’s leaftail gecko (*Uroplatus henkeli*); Hispaniolan masked curly-tailed lizard (*Leiocephalus personatus mentalis*); Leopard gecko (*Eublepharis macularius*); Little brown skink (*Scincella lateralis*); New Web-footed gecko (*Pachydactylus rangei)*; Ocellated skink (*Chalcides ocellatus*); Short skink (*Tiliqua rugosa*); Shreiber’s curly tailed lizard (*Leiocephalus eremitus*); Small head worm lizard (*Leposternon microcephalum*); Tanganyika wedge-snouted worm lizard (*Geocalamus acutus*); Texas Horned Lizard (*Phrynosoma cornutum)*;
Emotion	Explored	Green iguana (*Iguana iguana*); Wood turtle (*Glyptemys insculpta*)
Excitement	Assumed	Green iguana (*Iguana iguana*)
Fear	Assumed	Brown basilisk (*Basiliscus vittatus*); Common basilisk (*Basiliscus basiliscus*); El Hierro giant lizard (*Gallotia simonyi*); Iberian wall lizard (*Podarcis hispanicus)*; Many-striped skink (*Plestiodon multivirgatus)*; Schneider’s skink (*Eumeces schneiderii*)
Frustration	Assumed	Ball python (*Python regius*)
Pain	Assumed	American alligator (*Alligator mississippiensis*); Australian freshwater crocodiles (*Crocodylus johnsoni*); Ball python (*Python regius*); Bearded dragon (*Pogona vitticeps*); Estuarine crocodile (*Crocodylus porosus*) (x2); Fly river turtle (*Carettochelys insculpta*); Galloti lizard (*Gallotia galloti*); Green iguana (*Iguana iguana*); Green lizards (*Lacerta bilineata*); Horsfield’s tortoises (*Testudo horsfieldii*); Loggerhead sea turtle (*Caretta caretta*) (x3); Macquarie river turtle (*Emydura macquarii*); Red-eared slider (*Trachemys scripta elegans*) (x3); Timber rattlesnake (*Crotalus horrideus*); Woma python (*Aspidites ramsayi*); Yellow-bellied slider turtle (*Trachemys scripta scripta*) (x2)
Pleasure	Explored	Brown basilisk (*Basiliscus vittatus*); Common basilisk (*Basiliscus basiliscus*); Many-striped skink (*Plestiodon multivirgatus)*; Schneider’s skink (*Eumeces schneiderii*)
Stress	Assumed	Green anole (*Anolis carolinensis)*; Eastern blue tongued lizard (*Tiliqua scincoides scincoides)*; Brown basilisk (*Basiliscus vittatus*); Common basilisk (*Basiliscus basiliscus*); Many-striped skink (*Plestiodon multivirgatus)*; Schneider’s skink (*Eumeces schneiderii*); Wood turtle (*Glyptemys insculpta*)
Suffering	Assumed	Japanese Gecko (*Gekko japonicus*)

**Table 4:** Species that were studied in more than one article.

Species	Number of Research Articles Species Was Studied in
Red-eared slider turtles (*Trachemys scripta elegans*)	3
Bearded dragon (*Pogona vitticeps*)	2
Green iguana (*Iguana iguana*)	2
Loggerhead sea turtle (*Caretta caretta*)	3
Estuarine crocodile (*Crocodylus porosus*)	2
Ball python (*Python regius*)	2
Green anole (*Anolis carolinensis)*	2
Yellow-bellied slider turtle (*Trachemys scripta scripta*)	2
Wood turtle (*Glyptemys insculpta*)	2

## Appendix A

**Table A1:** The keywords used in the study, including details of their source, valence and whether they returned suitable results.

Keyword	Origin	Valence	Returned Results
Awe	Plutchik (1981), Proctor et al. (2013)	Positive	No
Amazement	Plutchik (1981), Parrot (2001), Proctor et al. (2013)	Positive	No
Admiration	Plutchik (1981), Proctor et al. (2013)	Positive	No
Acceptance	Plutchik (1981), Proctor et al. (2013)	Neutral	No
Apprehension	Plutchik (1981), Parrot (2001), Proctor et al. (2013)	Negative	No
Annoyance	Plutchik (1981), Parrot (2001), HUMAINE (2006), Proctor et al. (2013)	Negative	No
Anticipation	Plutchik (1981), Proctor et al. (2013)	Neutral	No
Anger	Plutchik (1981), Parrot (2001), HUMAINE (2006), Proctor et al. (2013)	Negative	No
Affection	Parrot (2001), Proctor et al. (2013)	Positive	No
Adoration	Parrot (2001), Proctor et al. (2013)	Positive	No
Attraction	Parrot (2001), Proctor et al. (2013)	Positive	No
Arousal (emotional)	Parrot (2001), Proctor et al. (2013)	Neutral	No
Amusement	Parrot (2001), Proctor et al. (2013)	Positive	No
Astonishment	Parrot (2001), Proctor et al. (2013)	Neutral	No
Aggravation	Parrot (2001), Proctor et al. (2013)	Negative	No
Agitation	Parrot (2001), Proctor et al. (2013)	Negative	No
Agony	Parrot (2001), Proctor et al. (2013)	Negative	No
Anguish	Parrot (2001), Proctor et al. (2013)	Negative	No
Alienation	Parrot (2001), Proctor et al. (2013)	Negative	No
Alarm	Parrot (2001), Proctor et al. (2013)	Negative	No
Anxiety	Parrot (2001), HUMAINE (2006), Proctor et al. (2013)	Negative	Yes
Altruism	Proctor et al. (2013)	Positive	No
Affective State	Proctor et al. (2013)	Neutral	No
Boredom	Plutchik (1981), HUMAINE (2006), Proctor et al. (2013)	Negative	No
Bliss	Parrot (2001), Proctor et al. (2013)	Positive	No
Bitterness	Parrot (2001), Proctor et al. (2013)	Negative	No
Contempt	Plutchik (1981), Parrot (2001), HUMAINE (2006), Proctor et al. (2013)	Negative	No
Caring	Parrot (2001), Proctor et al. (2013)	Positive	No
Compassion	Parrot (2001), Proctor et al. (2013)	Positive	No
Cheerfulness	Parrot (2001), Proctor et al. (2013)	Positive	No
Contentment	Parrot (2001), Proctor et al. (2013)	Positive	No
Conscious	Proctor et al. (2013)	Neutral	No
Cognitive Ethology	Proctor et al. (2013)	Neutral	No
Cognitive bias	New	Neutral	No
Disapproval	Plutchik (1981), Proctor et al. (2013)	Negative	No
Distraction	Plutchik (1981), Proctor et al. (2013)	Neutral	No
Disgust	Plutchik (1981), Parrot (2001), HUMAINE (2006), Proctor et al. (2013)	Negative	No
Desire	Parrot (2001), Proctor et al. (2013)	Neutral	No
Delight	Parrot (2001), Proctor et al. (2013)	Positive	No
Dislike	Parrot (2001), Proctor et al. (2013)	Negative	No
Depression	Parrot (2001), Proctor et al. (2013)	Negative	No
Despair	Parrot (2001), HUMAINE (2006), Proctor et al. (2013)	Negative	No
Dismay	Parrot (2001, Proctor et al. (2013)	Negative	No
Disappointment	Parrot (2001), HUMAINE (2006), Proctor et al. (2013)	Negative	No
Displeasure	Parrot (2001), Proctor et al. (2013)	Negative	No
Defeat	Parrot (2001), Proctor et al. (2013)	Negative	No
Dejection	Parrot (2001), Proctor et al. (2013)	Negative	No
Distress	Parrot (2001), Proctor et al. (2013)	Negative	Yes
Dread	Parrot (2001), Proctor et al. (2013)	Negative	No
Doubt	HUMAINE (2006), Proctor et al. (2013)	Negative	No
Ecstasy	Plutchik (1981), Parrot (2001), Proctor et al. (2013)	Positive	No
Enjoyment	Parrot (2001), Proctor et al. (2013)	Positive	No
Elation	Parrot (2001), Proctor et al. (2013)	Positive	No
Euphoria	Parrot (2001), Proctor et al. (2013)	Positive	No
Enthusiasm	Parrot (2001), Proctor et al. (2013)	Positive	No
Excitement	Parrot (2001), Proctor et al. (2013)	Positive	Yes
Exhilaration	Parrot (2001), Proctor et al. (2013)	Positive	No
Eagerness	Parrot (2001), Proctor et al. (2013)	Positive	No
Enthrallment	Parrot (2001), Proctor et al. (2013)	Positive	No
Exasperation	Parrot (2001), Proctor et al. (2013)	Negative	No
Envy	Parrot (2001), HUMAINE (2006), Proctor et al. (2013)	Negative	No
Embarrassment	Parrot (2001), HUMAINE (2006), Proctor et al. (2013)	Negative	No
Empathy	Proctor et al. (2013)	Neutral	No
Emotion	Proctor et al. (2013)	Neutral	Yes
Fear	Plutchik (1981), Parrot (2001), HUMAINE (2006)	Negative	Yes
Fondness	Parrot (2001), Proctor et al. (2013)	Positive	
Frustration	Parrot (2001), HUMAINE (2006), Proctor et al. (2013)	Negative	Yes
Fury	Parrot (2001), Proctor et al. (2013)	Negative	No
Ferocity	Parrot (2001), Proctor et al. (2013)	Negative	No
Fright	Parrot (2001), Proctor et al. (2013)	Negative	No
Grief	Plutchik (1981), Parrot (2001), Proctor et al. (2013)	Negative	No
Gaiety	Parrot (2001), Proctor et al. (2013)	Positive	No
Glee	Parrot (2001), Proctor et al. (2013)	Positive	No
Gladness	Parrot (2001), Proctor et al. (2013)	Positive	No
Grouchiness	Parrot (2001), Proctor et al. (2013)	Negative	No
Grumpiness	Parrot (2001), Proctor et al. (2013)	Negative	No
Gloom	Parrot (2001), Proctor et al. (2013)	Negative	No
Glumness	Parrot (2001), Proctor et al. (2013)	Negative	No
Guilt	Parrot (2001), HUMAINE (2006), Proctor et al. (2013)	Negative	No
Generosity	Proctor et al. (2013)	Positive	No
Happiness	Parrot (2001), Proctor et al. (2013)	Positive	No
Hope	Parrot (2001), Proctor et al. (2013)	Positive	No
Hostility	Parrot (2001), Proctor et al. (2013)	Negative	No
Hate	Parrot (2001), Proctor et al. (2013)	Negative	No
Hopelessness	Parrot (2001), Proctor et al. (2013)	Negative	No
Homesickness	Parrot (2001), Proctor et al. (2013)	Negative	No
Humiliation	Parrot (2001), Proctor et al. (2013)	Negative	No
Horror	Parrot (2001), Proctor et al. (2013)	Negative	No
Hysteria	Parrot (2001), Proctor et al. (2013)	Negative	No
Helplessness	HUMAINE (2006), Proctor et al. (2013)	Negative	No
Hurt	HUMAINE (2006, Proctor et al. (2013))	Negative	No
Infatuation	Parrot (2001), Proctor et al. (2013)	Neutral	No
Irritation	Parrot (2001), HUMAINE (2006), Proctor et al. (2013)	Negative	No
Isolation	Parrot (2001), Proctor et al. (2013)	Negative	No
Insecurity	Parrot (2001), Proctor et al. (2013)	Negative	No
Insult	Parrot (2001), Proctor et al. (2013)	Negative	No
Interest	Plutchik (1981), Proctor et al. (2013)	Neutral	No
Joy	Parrot (2001), Plutchik (1981), Proctor et al. (2013)	Positive	No
Jolliness	Parrot (2001), Proctor et al. (2013)	Positive	No
Joviality	Parrot (2001), Proctor et al. (2013)	Positive	No
Jubilation	Parrot (2001), Proctor et al. (2013)	Positive	No
Jealousy	Parrot (2001), Proctor et al. (2013)	Negative	No
Judgement bias	New	Neutral	No
Love	Parrot (2001), Plutchik (1981), Proctor et al. (2013)	Positive	No
Loathing	Parrot (2001), Plutchik (1981), Proctor et al. (2013)	Negative	No
Liking	Parrot (2001), Proctor et al. (2013)	Positive	No
Lust	Parrot (2001), Proctor et al. (2013)	Positive	No
Longing	Parrot (2001), Proctor et al. (2013)	Negative	No
Loneliness	Parrot (2001), Proctor et al. (2013)	Negative	No
Misery	Parrot (2001), Proctor et al. (2013)	Negative	No
Melancholy	Parrot (2001), Proctor et al. (2013)	Negative	No
Mortification	Parrot (2001), Proctor et al. (2013)	Negative	No
Morality	Proctor et al. (2013)	Neutral	No
Mourn	Proctor et al. (2013)	Negative	No
Modest	Proctor et al. (2013)	Neutral	No
Neglect	Parrot (2001), Proctor et al. (2013)	Negative	No
Nervousness	Parrot (2001), Proctor et al. (2013)	Negative	No
Optimism	Parrot (2001), Plutchik (1981), Proctor et al. (2013)	Positive	No
Outrage	Parrot (2001), Proctor et al. (2013)	Negative	No
Pensiveness	Plutchik (1981), Proctor et al. (2013)	Neutral	No
Passion	Parrot (2001), Proctor et al. (2013)	Positive	No
Pleasure	Parrot (2001), Proctor et al. (2013)	Positive	Yes
Pride	Parrot (2001), Proctor et al. (2013)	Positive	No
Pity	Parrot (2001), Proctor et al. (2013)	Negative	No
Panic	Parrot (2001), Proctor et al. (2013)	Negative	No
Powerlessness	HUMAINE (2006), Proctor et al. (2013)	Negative	No
Pessimism	Proctor et al. (2013)	Negative	No
Play	Proctor et al. (2013)	Positive	No
Pain	Proctor et al. (2013)	Negative	Yes
Rage	Parrot (2001), Plutchik (1981), Proctor et al. (2013)	Negative	No
Remorse	Parrot (2001), Plutchik (1981), Proctor et al. (2013)	Negative	No
Rapture	Parrot (2001), Proctor et al. (2013)	Negative	No
Relief	Parrot (2001), Proctor et al. (2013)	Positive	No
Resentment	Parrot (2001), Proctor et al. (2013)	Negative	
Revulsion	Parrot (2001), Proctor et al. (2013)	Negative	No
Regret	Parrot (2001), Proctor et al. (2013)	Negative	No
Rejection	Parrot (2001), Proctor et al. (2013)	Negative	No
Revenge	Proctor et al. (2013)	Negative	No
Rationality	Proctor et al. (2013)	Neutral	No
Surprise	Plutchik (1981), Proctor et al. (2013)	Neutral	No
Sadness	Plutchik (1981), Parrot (2001)	Negative	No
Submission	Plutchik (1981), Proctor et al. (2013)	Neutral	
Serenity	Plutchik (1981), Proctor et al. (2013)	Positive	No
Shock	Parrot (2001)	Negative	No
Sentience	Proctor et al. (2013)	Neutral	No
Sentimentality	Parrot (2001), Proctor et al. (2013)	Neutral	No
Satisfaction	Parrot (2001), Proctor et al. (2013)	Positive	No
Scorn	Parrot (2001), Proctor et al. (2013)	Negative	No
Spite	Parrot (2001), Proctor et al. (2013)	Negative	No
Suffering	Parrot (2001), Proctor et al. (2013)	Negative	Yes
Shame	Parrot (2001), HUMAINE (2006), Proctor et al. (2013)	Negative	No
Sorrow	Parrot (2001), Proctor et al. (2013)	Negative	No
Sympathy	Parrot (2001), Proctor et al. (2013)	Neutral	No
Shock	Parrot (2001), Proctor et al. (2013)	Negative	No
Sentience	Proctor et al. (2013)	Neutral	No
Stress	Proctor et al. (2013)	Negative	Yes
Trust	Plutchik (1981), Proctor et al. (2013)	Positive	No
Terror	Plutchik (1981), Parrot (2001)	Negative	No
Tenderness	Parrot (2001), Proctor et al. (2013)	Positive	No
Thrill	Parrot (2001), Proctor et al. (2013)	Positive	No
Triumph	Parrot (2001), Proctor et al. (2013)	Positive	No
Torment	Parrot (2001), Proctor et al. (2013)	Negative	No
Tenseness	Parrot (2001), Proctor et al. (2013)	Negative	No
Unhappiness	Parrot (2001), Proctor et al. (2013)	Negative	No
Uneasiness	Parrot (2001), Proctor et al. (2013)	Negative	No
Vigilance	Plutchik (1981), Proctor et al. (2013)	Neutral	No
Vengefulness	Parrot (2001), Proctor et al. (2013)	Negative	No
Valence	Proctor et al. (2013)	Neutral	No
Wrath	Parrot (2001), Proctor et al. (2013)	Negative	No
Woe	Parrot (2001), Proctor et al. (2013)	Negative	No
Worry	Parrot (2001), HUMAINE (2006), Proctor et al. (2013)	Negative	No
Zeal	Parrot (2001), Proctor et al. (2013)	Positive	No
Zest	Parrot (2001), Proctor et al. (2013)	Negative	No

**Table A2:** The articles found assuming the sentience or cognition keywords.

Keyword	Article
Anxiety	Li, J., Wang, Y., Li, W., Xu, P., Guo, B., Li, J., and Wang, H. (2017). Tissue distribution and metabolism of triadimefon and triadimenol enantiomers in Chinese lizards (Eremias argus). *Ecotoxicology and Environmental Safety*, *142*, 284–292.
Anxiety	Lima-Maximino, M. G., Cueto-Escobedo, J., Rodríguez-Landa, J. F., and Maximino, C. (2018). FGIN-1-27, an agonist at translocator protein 18 kDa (TSPO), produces anti-anxiety and anti-panic effects in non-mammalian models. *Pharmacology Biochemistry and Behavior*, *171*, 66–73.
Anxiety	Paitz, R. T., Clairardin, S. G., Gould, A. C., Hicke, J. W., Zimmerman, L. M., and Bowden, R. M. (2014). Corticosterone levels during the nesting process in red-eared sliders (Trachemys scripta). *Journal of Herpetology*, *48*(4), 567–570.
Distress	Sherbrooke, W. C. (2008). Antipredator responses by Texas horned lizards to two snake taxa with different foraging and subjugation strategies. *Journal of Herpetology*, *42*(1), 145–153.
Distress	Wassersug, R. J., Roberts, L., Gimian, J., Hughes, E., Saunders, R., Devison, D., …, and O’Reilly, J. C. (2005). The behavioral responses of amphibians and reptiles to microgravity on parabolic flights. *Zoology*, *108*(2), 107–120.
Excitement	Mosley, C. A., Dyson, D., and Smith, D. A. (2004). The cardiovascular dose–response effects of isoflurane alone and combined with butorphanol in the green iguana (Iguana iguana). *Veterinary Anaesthesia and Analgesia*, *31*(1), 64–72.
Fear	Burunat-Pérez, G., Suárez-Rancel, M., and Molina-Borja, M. (2018). Predator avoidance training of the endangered lizard from El Hierro (Canary Islands): A new management strategy before reintroduction into the wild. *Behavioural processes*, *157*, 192–198.
Fear	Davies, D. C., Martınez-Garcıa, F., Lanuza, E., and Novejarque, A. (2002). Striato-amygdaloid transition area lesions reduce the duration of tonic immobility in the lizard Podarcis hispanica. *Brain research bulletin*, *57*(3-4), 537–541.
Fear	Paradis, S., and Cabanac, M. (2004). Flavor aversion learning induced by lithium chloride in reptiles but not in amphibians. *Behavioural processes*, *67*(1), 11–18.
Frustration	Rose, M. P., and Williams, D. L. (2014). Neurological dysfunction in a ball python (Python regius) colour morph and implications for welfare. *Journal of Exotic Pet Medicine*, *23*(3), 234–239.
Pain	Bertelsen, M. F., and Sauer, C. D. (2011). Alfaxalone anaesthesia in the green iguana (Iguana iguana). *Veterinary anaesthesia and analgesia*, *38*(5), 461–466.
Pain	Couture, É. L., Monteiro, B. P., Aymen, J., Troncy, E., and Steagall, P. V. (2017). Validation of a thermal threshold nociceptive model in bearded dragons (Pogona vitticeps). *Veterinary anaesthesia and analgesia*, *44*(3), 676–683.
Pain	Delgado-Gonzalez, F. J., Gonzalez-Granero, S., Trujillo-Trujillo, C. M., Garcia-Verdugo, J. M., and Damas-Hernandez, M. C. (2011). Study of adult neurogenesis in the Gallotia galloti lizard during different seasons. *Brain research*, *1390*, 50–58.
Pain	Giorgi, M., Rota, S., Giorgi, T., Capasso, M., and Briganti, A. (2013). Blood concentrations of enrofloxacin and the metabolite ciprofloxacin in yellow-bellied slider turtles (Trachemys scripta scripta) after a single intracoelomic injection of enrofloxacin. *Journal of exotic pet medicine*, *22*(2), 192–199.
Pain	Giorgi, M., Lee, H. K., Rota, S., Owen, H., De Vito, V., Demontis, M. P., and Varoni, M. V. (2015). Pharmacokinetic and pharmacodynamic assessments of tapentadol in yellow-bellied slider turtles (Trachemys scripta scripta) after a single intramuscular injection. *Journal of Exotic Pet Medicine*, *24*(3), 317–325.
Pain	Gregorovičová, M., and Černíková, A. (2015). Reactions of green lizards (Lacerta viridis) to major repellent compounds secreted by Graphosoma lineatum (Heteroptera: Pentatomidae). *Zoology*, *118*(3), 176-182.
Pain	Hale, V. L., MacGowan, B., Corriveau, L., Huse, D. C., Currylow, A. F., and Thompson, S. (2017). Radio transmitter implantation and movement in the wild timber rattlesnake (Crotalus horridus). *Journal of wildlife diseases*, *53*(3), 591–595.
Pain	Hansen, L. L., and Bertelsen, M. F. (2013). Assessment of the effects of intramuscular administration of alfaxalone with and without medetomidine in Horsfield’s tortoises (A grionemys horsfieldii). *Veterinary anaesthesia and analgesia*, *40*(6), e68–e75.
Pain	Hartzler, L. K., Munns, S. L., Bennett, A. F., and Hicks, J. W. (2006). Recovery from an activity-induced metabolic acidosis in the American alligator, Alligator mississippiensis. *Comparative Biochemistry and Physiology Part A: Molecular & Integrative Physiology*, *143*(3), 368–374.
Pain	James, L. E., Williams, C. J., Bertelsen, M. F., and Wang, T. (2017). Evaluation of feeding behavior as an indicator of pain in snakes. *Journal of zoo and wildlife medicine*, *48*(1), 196–199.
Pain	Johnson, S. M., Moris, C. M., Bartman, M. E., and Wiegel, L. M. (2010). Excitatory and inhibitory effects of opioid agonists on respiratory motor output produced by isolated brainstems from adult turtles (Trachemys). *Respiratory physiology & neurobiology*, *170*(1), 5–15.
Pain	Kaye, S. W., Daverio, H., Eddy, R., Ossiboff, R. J., Peters-Kennedy, J., and Morrisey, J. K. (2016). Surgical Resection of an Interrenal Cell Adenocarcinoma in a Woma Python (Aspidites ramsayi) with 18 Month Follow-up. *Journal of Herpetological Medicine and Surgery*, *26*(1–2), 26–31.
Pain	Kinney, M. E., Johnson, S. M., and Sladky, K. K. (2011). Behavioral evaluation of red-eared slider turtles (Trachemys scripta elegans) administered either morphine or butorphanol following unilateral gonadectomy. *Journal of Herpetological Medicine and Surgery*, *21*(2), 54–62.
Pain	Lai, O. R., Di Bello, A., Soloperto, S., Freggi, D., Marzano, G., Cavaliere, L., and Crescenzo, G. (2015). Pharmacokinetic behavior of meloxicam in loggerhead sea turtles (Caretta caretta) after intramuscular and intravenous administration. *Journal of wildlife diseases*, *51*(2), 509–512.
Pain	Manire, C. A., Hunter, R. P., Koch, D. E., Byrd, L., and Rhinehart, H. L. (2005). Pharmacokinetics of ticarcillin in the loggerhead sea turtle (Caretta caretta) after single intravenous and intramuscular injections. *Journal of Zoo and Wildlife Medicine*, *36*(1), 44–54.
Pain	Morici, M., Interlandi, C., Costa, G. L., Di Giuseppe, M., and Spadola, F. (2017). Sedation with intracloacal administration of dexmedetomidine and ketamine in yellow-bellied sliders (Trachemys scripta scripta). *Journal of exotic pet medicine*, *26*(3), 188–191.
Pain	Norton, T. M., Cox, S., Nelson, S. E., Jr., Kaylor, M., Thomas, R., Hupp, A., and Sladky, K. K. (2015). Pharmacokinetics of tramadol and O-desmethyltramadol in loggerhead sea turtles (Caretta caretta). *Journal of Zoo and Wildlife Medicine*, *46*(2), 262–265.
Pain	Olsson, A., Phalen, D., and Dart, C. (2013). Preliminary studies of alfaxalone for intravenous immobilization of juvenile captive estuarine crocodiles (Crocodylus porosus) and Australian freshwater crocodiles (Crocodylus johnstoni) at optimal and selected sub-optimal thermal zones. *Veterinary anaesthesia and analgesia*, *40*(5), 494–502.
Pain	Olsson, A., and Phalen, D. (2013). The effects of decreased body temperature on the onset, duration and action of medetomidine and its antagonist atipamezole in juvenile farmed estuarine crocodiles (Crocodylus porosus). *Veterinary anaesthesia and analgesia*, *40*(3), 272–279.
Pain	Scheelings, T. F. (2013). Use of intravenous and intramuscular alfaxalone in Macquarie river turtles (Emydura macquarii). *Journal of Herpetological Medicine and Surgery*, *23*(3), 91–94.
Pain	Shepard, M. K., Divers, S., Braun, C., and Hofmeister, E. H. (2013). Pharmacodynamics of alfaxalone after single-dose intramuscular administration in red-eared sliders (Trachemys scripta elegans): a comparison of two different doses at two different ambient temperatures. *Veterinary anaesthesia and analgesia*, *40*(6), 590–598.
Pain	Tuxbury, K. A., Clayton, L. A., Snakard, E. P., and Fishman, E. K. (2010). Multiple skull fractures in a captive fly river turtle (Carretochelys insculpta): diagnosis, surgical repair, and medical management. *Journal of Herpetological Medicine and Surgery*, *20*(1), 11–19.
Stress	Borgmans, G., Palme, R., Sannen, A., Vervaecke, H., and Van Damme, R. (2018). The effect of environmental provisioning on stress levels in captive green anole (Anolis carolinensis). *Animal Welfare*, *27*(1), 35–46.
Stress	Cabanac, M., and Bernieri, C. (2000). Behavioral rise in body temperature and tachycardia by handling of a turtle (Clemmys insculpta). *Behavioural Processes*, *49*(2), 61–68.
Stress	Mancera, K., Murray, P. J., Gao, Y. N., Lisle, A., and Phillips, C. J. C. (2014). The effects of simulated transport on the behaviour of eastern blue tongued lizards (Tiliqua scincoides). *Animal Welfare*, *23*(3), 239–249.
Stress	Paradis, S., and Cabanac, M. (2004). Flavor aversion learning induced by lithium chloride in reptiles but not in amphibians. *Behavioural processes*, *67*(1), 11–18.
Suffering	Li, J., Wu, R., Chen, H., Zhou, Y., Li, Y., Wang, Y., …, and Liu, M. (2013). The cloning and characterization of the Enolase2 gene of Gekko japonicus and its polyclonal antibody preparation. *International journal of molecular sciences*, *14*(5), 8787–8800.
